# High seroprevalence of Leishmania infantum is linked to immune activation in people with HIV: a two-stage cross-sectional study in Bahia, Brazil

**DOI:** 10.3389/fmicb.2023.1221682

**Published:** 2023-07-19

**Authors:** Laise de Moraes, Luciane Amorim Santos, Liã Bárbara Arruda, Maria da Purificação Pereira da Silva, Márcio de Oliveira Silva, José Adriano Góes Silva, André Ramos, Marcos Bastos dos Santos, Felipe Guimarães Torres, Cibele Orge, Antonio Marcos dos Santos Teixeira, Thiago Santos Vieira, Laura Ramírez, Manuel Soto, Maria Fernanda Rios Grassi, Isadora Cristina de Siqueira, Dorcas Lamounier Costa, Carlos Henrique Nery Costa, Bruno de Bezerril Andrade, Kevan Akrami, Camila Indiani de Oliveira, Viviane Sampaio Boaventura, Manoel Barral-Netto, Aldina Barral, Anne-Mieke Vandamme, Johan Van Weyenbergh, Ricardo Khouri

**Affiliations:** ^1^Programa de Pós-graduação em Ciências da Saúde, Faculdade de Medicina da Bahia, Universidade Federal da Bahia, Salvador, Brazil; ^2^Instituto Gonçalo Moniz, Fundação Oswaldo Cruz, Salvador, Brazil; ^3^Escola Bahiana de Medicina e Saúde Pública, Salvador, Brazil; ^4^Centre for Clinical Microbiology, Division of Infection & Immunity, University College London, London, United Kingdom; ^5^Centro Estadual Especializado em Diagnóstico, Assistência e Pesquisa, Secretaria de Saúde do Estado da Bahia, Salvador, Brazil; ^6^Departamento de Biología Molecular, Facultad de Ciencias, Centro de Biología Molecular Severo Ochoa, Consejo Superior de Investigaciones Científicas, Universidad Autónoma de Madrid, Madrid, Spain; ^7^Laboratório de Leishmanioses, Instituto de Doenças Tropicais Natan Portella, Universidade Federal do Piauí, Teresina, Brazil; ^8^Hospital Santa Izabel, Salvador, Brazil; ^9^Department of Microbiology, Immunology and Transplantation, Rega Institute for Medical Research, Clinical and Epidemiological Virology, Leuven, Belgium; ^10^Center for Global Health and Tropical Medicine, Instituto de Higiene e Medicina Tropical, Universidade Nova de Lisboa, Lisbon, Portugal

**Keywords:** HIV-1, Leishmania infantum, visceral leishmaniasis, immune activation, hostpathogen interaction

## Abstract

Visceral leishmaniasis is an opportunistic disease in HIV-1 infected individuals, unrecognized as a determining factor for AIDS diagnosis. The growing geographical overlap of HIV-1 and *Leishmania* infections is an emerging challenge worldwide, as co-infection increases morbidity and mortality for both infections. Here, we determined the prevalence of people living with HIV (PWH) with a previous or ongoing infection by *Leishmania infantum* and investigated the virological and immunological factors associated with co-infection. We adopted a two-stage cross-sectional cohort (CSC) design (CSC-I, *n* = 5,346 and CSC-II, *n* = 317) of treatment-*naïve* HIV-1-infected individuals in Bahia, Brazil. In CSC-I, samples collected between 1998 and 2013 were used for serological screening for leishmaniasis by an in-house Enzyme-Linked Immunosorbent Assay (ELISA) with SLA (Soluble *Leishmania infantum* Antigen), resulting in a prevalence of previous or ongoing infection of 16.27%. Next, 317 PWH were prospectively recruited from July 2014 to December 2015 with the collection of sociodemographic and clinical data. Serological validation by two different immunoassays confirmed a prevalence of 15.46 and 8.20% by anti-SLA, and anti-HSP70 serology, respectively, whereas 4.73% were double-positive (DP). Stratification of these 317 individuals in DP and double-negative (DN) revealed a significant reduction of CD4^+^ counts and CD4^+^/CD8^+^ ratios and a tendency of increased viral load in the DP group, as compared to DN. No statistical differences in HIV-1 subtype distribution were observed between the two groups. However, we found a significant increase of CXCL10 (*p* = 0.0076) and a tendency of increased CXCL9 (*p* = 0.061) in individuals with DP serology, demonstrating intensified immune activation in this group. These findings were corroborated at the transcriptome level in independent Leishmania- and HIV-1-infected cohorts (Swiss HIV Cohort and Piaui Northeast Brazil Cohort), indicating that CXCL10 transcripts are shared by the IFN-dominated immune activation gene signatures of both pathogens and positively correlated to viral load in untreated PWH. This study demonstrated a high prevalence of PWH with *L. infantum* seropositivity in Bahia, Brazil, linked to IFN-mediated immune activation and a significant decrease in CD4^+^ levels. Our results highlight the urgent need to increase awareness and define public health strategies for the management and prevention of HIV-1 and *L. infantum* co-infection.

## 1. Introduction

HIV is the etiological agent of Acquired Immunodeficiency Syndrome (AIDS), a slowly progressive disease characterized by chronic immune activation resulting from the loss of cell-mediated immune function (Costin, 2007; Perkins et al., 2015). Host genetic factors (e.g., HLA genotypes) and host immune response (e.g., release of proinflammatory cytokines and chemokines), as well as the presence of co-infections, can directly influence the rate of disease progression (Claiborne et al., 2015; Kouri et al., 2015; Perkins et al., 2015).

Neglected Tropical Diseases (NTDs) are endemic among the most impoverished populations in Africa, Asia and Latin America. Among these NTDs, visceral leishmaniasis (VL), caused by infection with *Leishmania infantum*, is associated with increased morbidity and mortality in people with HIV-1 (PWH) but has not been recognized as a diagnostic criterion for AIDS (van Griensven et al., 2014). The World Health Organization (WHO) identified the geographical overlap of both infections as an emerging challenge in successfully controlling the HIV-1 infection in countries considered endemic for leishmaniasis, such as Brazil (World Health Organization, 2015).

The few studies evaluating the prevalence of HIV-1 and *L. infantum* coinfection on a national level in Brazil (Sousa-Gomes et al., 2011; Coura-Vital et al., 2014) have highlighted regions endemic for leishmaniasis: Distrito Federal (Carranza-Tamayo et al., 2009), Ceará (Távora et al., 2015), Maranhão (Carvalho et al., 2013), Mato Grosso (Luz et al., 2018), Mato Grosso do Sul (Botelho and Natal, 2009), Minas Gerais (Souza et al., 2012; Cota et al., 2014), Pernambuco (Sousa et al., 2018), Piauí (Soares et al., 2008), Rio Grande do Norte (Lima et al., 2017), Sergipe (Santos et al., 2019), and Tocantins (Albuquerque et al., 2014). Bahia is also considered an endemic area for leishmaniasis, with an incidence rate of 0.8/100,000 inhabitants for VL reported in 2022 (Secretaria de Saúde do Estado da Bahia, 2022b). In addition, the increase of geographical overlap between HIV-1 and *Leishmania* infection in Bahia (Secretaria de Saúde do Estado da Bahia, 2022a, 2022b) has raised public health concerns. However, no epidemiological studies have attempted to investigate the prevalence of co-infection to date. Therefore, we investigated the seroprevalence of *L. infantum* in PWH in the state of Bahia, Brazil and attempted to identify virological and immunological factors associated with co-infection, supported by publicly available transcriptomic data.

## 2. Methods

### 2.1. Study population and design

**Figure 1:**
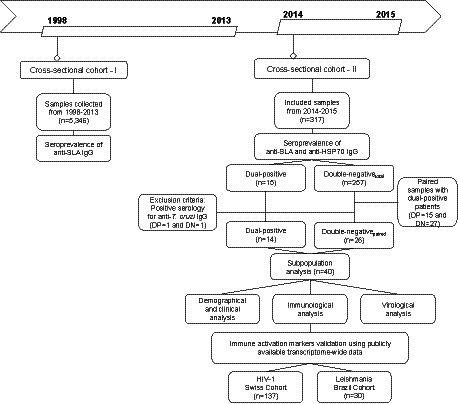
Flow chart of study design detailing the two-stage cross-sectional and prospective cohort study among people living with HIV in Bahia, Brazil.

A two-stage cross-sectional cohort (CSC) study (CSC-I, 1998–2013 and CSC-II, 2014–2015) was designed to determine the seroprevalence of *L. infantum* in PWH and to evaluate the association with demographic, clinical, virological, and immunological parameters (Figure 1). From 1998 to 2015, individuals with a new confirmed diagnosis of HIV-1 at the Specialized Center for Diagnosis, Care and Research (CEDAP), a state government public health reference service located in the city of Salvador, Bahia-Brazil, were included in this study. Their remaining blood sample was utilized for the outlined assays herein.

First, serological reactivity to *L. infantum* was assessed in plasma or serum samples from the retrospective cross-sectional cohort (*n* = 5,346) collected between 1998 and 2013. In the second stage, serological reactivity to *L. infantum* was assessed in a prospective cross-sectional cohort of 317 PWH (sampled between July 2014 and December 2015), with demographic and clinical data (sex, age, HIV-1 viral load, CD4^+^, CD8^+^, CD4^+^/CD8^+^ ratio and CD45^+^ T cell counts) prospectively obtained from the clinical records. Additionally, we performed a subpopulation analysis on a subset of PWH identified as either double-positive or double-negative using two independent anti-*Leishmania* immunoassays. Based on these stringently defined categories, we compared virological and immunological factors influenced by *L. infantum* seropositivity. The study’s recruitment did not interfere with standard medical care, and patients were treated according to the general Brazilian/WHO guidelines. This study was conducted following the Declaration of Helsinki and was approved by the Institutional Review Board of the Gonçalo Moniz Institute (IGM-FIOCRUZ) (protocol number 1.764.505).

### 2.2. Detection of anti-*Leishmania* and anti-*Trypanosoma* antibodies

All samples underwent a previously described and validated in-house enzyme-linked immunosorbent assay (ELISA) for the detection of IgG against Soluble *Leishmania infantum* Antigen (SLA), which we previously demonstrated as a sensitive biomarker (90% sensitivity and 95% specificity) of current or past infection with the parasite (Charlab et al., 2000). SLA preparation and ELISA procedures were performed as previously described (Souza et al., 2013). For the prospective cohort of 317 samples from PWH with digitalized clinical records, results of the SLA immunoassay were compared with a second, previously described ELISA employing recombinant *L. infantum* heat shock protein 70 (HSP70), for which we once found a sensitivity of 74% and a specificity of 73% (Soto et al., 2015). To avoid false-positive results for *L. infantum* due to cross-reactivity among *Trypanosomatidae* parasites, an ELISA for anti-*Trypanosoma cruzi* IgG (Euroimmun) was employed in a subset of 42 samples with dual-positive (DP) (*n* = 15) and double-negative (*n* = 27) serology for anti-SLA and anti-HSP70.

### 2.3. Control samples and ELISA cutoff value

Positive (*n* = 6) *Leishmania*-infected patient samples and negative (*n* = 47) control samples previously identified by the intradermal Leishmanin (also known as Montenegro) Delayed-type Hypersensitivity (DTH) skin test (obtained from Fundação Ezequiel Dias, Belo Horizonte, Brazil), with clinical and laboratorial confirmation, were used to establish anti-SLA and anti-HSP70 ELISA cutoff values. For each assay, specific cutoff values were determined using the mean OD values obtained from the included negative samples plus three times the observed standard deviation, with each assay’s index values corresponding to the OD/cutoff ratio (Supplementary Figure S1).

### 2.4. Quantification of immunological markers

A Cytometric Bead Array (CBA) Human Inflammatory Cytokines Kit (BD Biosciences) was employed to quantify plasma concentrations of IL-1β, IL-6, IL-10, IL-12p70, and TNF, while CCL2/MPC-1, CCL5/RANTES, CXCL8/IL-8, CXCL9/MIG, and CXCL10/IP-10 quantification was performed using a CBA Human Chemokine Kit (BD Biosciences), each following the manufacturer’s instructions. Data acquisition and analysis were performed using a FACSArray Bioanalyzer (BD Biosciences) and FlowJo v10.0.5 (Tree Star) software, respectively.

### 2.5. HIV-1 sequencing analyses

Viral RNA isolation was performed using a QIAamp Viral RNA Mini Kit (QIAGEN) following the manufacturer’s instructions. The protease/reverse transcriptase (PR/RT) region was amplified and sequenced as previously described (Barreto et al., 2006). Outer polymerase chain reaction (PCR) was performed using a SuperScript III One-Step RT-PCR System with Platinum Taq DNA Polymerase (Thermo Fisher Scientific) and the following primers: K1 (CAGAGCCAACAGCCCCACC) and K2 (TTTCCCCACTAACTTCTGTATGTCATTGACA) (Kozal et al., 1996). Inner PCR was performed using Platinum Taq DNA Polymerase High Fidelity (Thermo Fisher Scientific) with the following primers: DP16 (CCTCAAATCACTCTTTGGCAAC) and RT4 (AGTTCATAACCCATCCAAAG) (Pieniazek et al., 1991). The generated inner PCR products were sequenced using a BigDye Terminator v.3.1 Cycle Sequencing Kit (Applied Biosystems) with capillary electrophoresis run on an ABI 3500xL Genetic Analyzer (Applied Biosystems) employing the following primers: F1 (GTTGACTCAGATTGGTTGCAC), F2 (GTATGTCATTGACAGTCCAGC) (Frenkel et al., 1995), DP10 (CAACTCCCTCTCAGAAGCAGGAGCCG), DP11 (CCATTCCTGGCTTTAATTTTACTGGTA) (Janini et al., 1996), RT4 (AGTTCATAACCCATCCAAAG), GABO1 (CTCARGACTTYTGGGAAGTTC), and GABO2 (GCATCHCCCACATCYAGTACTG) (Barreto et al., 2006).

Sequence visualization, editing and assembly were performed using Geneious v10.0.8 (Dotmatics) software. HIV-1 subtyping was determined using the REGA HIV-1 Subtyping Tool v.3.4.1^1^ and the jpHMM-HIV approach (Schultz et al., 2009). Phylogenetic analysis was conducted using HIV-1 subtype references retrieved from the Los Alamos HIV sequence database^2^ (Supplementary Table S1). Phylogenetic inference was performed using PhyML v3.0 (Guindon et al., 2010), applying the BIONJ method and using the General Time Reversible (GTR) nucleotide substitution model with 1,000 bootstrap replicates.

### 2.6. Transcriptome and systems biology analysis

Transcriptome analysis of publicly available data was performed as recently described (Vivarini et al., 2017; Delgobo et al., 2019; Subramanian et al., 2019). Overlap ratio was calculated as: k/K = # Genes in Overlap (k)/# Genes in Gene Set (K) using Molecular Signatures Database (MSigDb). HIV-1 (Swiss HIV Cohort, *n* = 137) (Rotger et al., 2010) and Leishmania-infected individuals (Piaui Northeast Brazil Cohort, *n* = 30) (Gardinassi et al., 2016) were analyzed using GEO2R from Gene Expression Omnibus (GEO).

### 2.7. Statistical analysis

Since data were not normally distributed, as identified by the Shapiro–Wilk and D’Agostino-Pearson tests (GraphPad Prism v7.0), results were analyzed using non-parametric tests: Mann–Whitney test and Spearman’s correlation, as well as Fisher’s exact test, with *p* < 0.05 considered statistically significant.

## 3. Results

### 3.1. Detection of IgG antibodies against *Leishmania infantum* antigens (anti-SLA and anti-HSP70) in people living with HIV-1

To determine the seroprevalence of *L. infantum* among PWHs, 5,346 plasma or serum samples from treatment-näive HIV-1-infected individuals diagnosed between 1998 and 2013 were tested for the presence of IgG antibodies against soluble *L. infantum* antigen (anti-SLA) (Figure 1).

**Figure 2:**
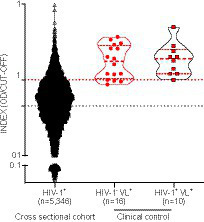
Detection of anti-*L. infantum* IgG antibodies in plasma/serum samples collected from PWH between 1998 and 2013. Violin plot with a dot-plot overlay of OD index values representative of *L. infantum* anti-SLA serology in the retrospective PWH cohort (*n* = 5,346) and a clinical- and laboratory-confirmed control group (HIV-1-VL+, *n* = 16; and HIV-1-VL+, *n* = 10). Filled black circles (●): PWH cross-sectional cohort samples; open triangles (Δ): clinical HIV-1 negative controls with VL; filled red squares with black borders (▪): clinical controls with HIV-1 positive controls and VL.

Positivity for anti-SLA serology was detected in 870/5,346 samples, resulting in a prevalence rate of 16.27% (Figure 2). We included additional positive control samples from patients with a clinically confirmed diagnosis of visceral leishmaniasis (VL) and laboratory-confirmed *L. infantum* infection in the absence (*n* = 16) or presence of HIV-1 co-infection (*n* = 10) (Zacarias et al., 2017). The detection rate of anti-SLA antibodies of the positive control samples was 81.25% for HIV-1^−^VL^+^ patients and 100% for HIV-1^+^VL^+^ patients (Figure 2). Besides, we found humoral immune response against SLA in VL patients quantitatively similar regardless of the presence of HIV-1 co-infection (Mann Whitney *p* = 0.71, HIV-negative vs. HIV-positive). Thus, we could confirm the high specificity of the *L. infantum* anti-SLA IgG serology protocol employed herein.

**Figure 3:**
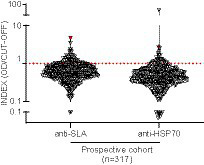
Detection of anti-*L. infantum* IgG antibodies in plasma samples collected from PWH between 2014 and 2015. Violin plot with a dot-plot overlay of OD index values representative of *L. infantum* anti-SLA and anti-HSP70 serology in the PWH prospective cohort (*n* = 317). The red symbols represent the DP individual diagnosed with visceral leishmaniasis >20 years ago.

Subsequently, to validate our findings, we enrolled an independent prospective CSC between 2014 and 2015, composed of 317 treatment-näive HIV-1-infected individuals from which complete demographic and clinical data were obtained from digitalized clinical records. Moreover, we combined anti-SLA serology with a second specific assay to detect IgG antibodies against *L. infantum* HSP70 recombinant protein (anti-HSP70) (Figure 1). The overall prevalence of anti-SLA positive serology in the prospective cohort was 15.46% (49/317), similar to the cross-sectional cohort results. The prevalence of anti-HSP70 positive serology in the prospective cohort was 8.20% (26/317) (Figure 3). Positivity for both anti-SLA and anti-HSP70 (i.e., double-positivity – DP) was detected in 4.73% (15/317) of the cohort; anti-SLA positive serology alone was detected in 10.73% (34/317); anti-HSP70 serology alone in only 3.47% (11/317) and the union of both anti-SLA and anti HSP70 positive serology was detected in 18.93% (Figure 3). Important, no statistical differences (Kruskal–Wallis test) were observed in viral load (*p* = 0.95), CD4^+^ (*p* = 0.93), CD8^+^ (*p* = 0.59), CD4^+^/CD8^+^ (*p* = 0.92), and CD45^+^ (*p* = 0.89) T cells values comparing DP, SLA, HSP70, and HSP70^+^SLA groups (Supplementary Table S6).

Based on these findings, DP (*n* = 15) was adopted as the most stringent criterium for *L. infantum* infection. Assessment of standard diagnosis of previous or ongoing visceral leishmaniasis (VL) documented in the clinical records showed that one of the 15 DP subjects had VL reported >20 years ago (anti-SLA index = 4.71; anti-HSP70 index = 2.67).

After two decades of clinical diagnosis, this pronounced humoral response most likely reflects chronic antigen exposure, supporting the concept that *Leishmania* infection is lifelong in most patients as observed in murine models of disease (Bogdan et al., 2000; Seyed et al., 2018; Paduch et al., 2019). These results, taken together, demonstrated that a significant number of PWH in the state of Bahia are unaware of their exposure to and/or infection by *L. infantum* and are at risk of developing new or relapsing visceral leishmaniasis.

### 3.2. Demographic and clinical markers associated with dual-positive *L. infantum* serology in PWH

The CSC-II (*n* = 317) consisted of predominantly young males (72.23%) with a median age of 32 years old at the time of HIV diagnosis, median viral load of 40,370 copies/mL (IQR = 7,032-141,359), CD4^+^ T cell count of 369 cells/μL (IQR = 163–609), CD8^+^ T cell count of 1,025 cells/μL (IQR = 715–1,441), and CD45^+^ T cell count of 2,144 cells/μL (IQR = 1,507–2,763) (Table 1).

**Table 1:** Epidemiological, virological, and immunological characteristics of PWH in Bahia (Brazil) between 2014 and 2,105, stratified according to DP and DN_total_.

	*n* = 317	DP (*n* = 15)	DN_total_ (*n* = 257)	*p* value
Male, *n*	72.23% (229)	73.33% (11)	70.42% (181)	NA
Age, years	32 (26–41)	41 (30–50)	32 (25–39)	**0.0430**
HIV-1 viral load, copies/mL	40,370 (7,032–141,359)	64,746 (2,480–532,702)	34,956 (6,330–127,747)	0.1268
CD4^+^ T cell count, cell/μL	369 (163–609)	168 (107–449)	405 (188–641)	**0.0208**
CD8^+^ T cell count, cell/μL	1,025 (715–1,441)	950 (688–1,546)	1,025 (728–1,443)	0.8679
CD4^+^/CD8^+^ T cell ratio	0.33 (0.14–0.59)	0.21 (0.060–0.37)	0.35 (0.15–0.63)	**0.0282**
CD45^+^ T cell count, cell/μL	2,144 (1,507–2,763)	1,758 (1,142–3,028)	2,198 (1,585–2,793)	0.3262

Data are expressed as median values, interquartile ranges (IIQ) and numbers of individuals evaluated (N), or as proportions (%) with the number of individuals evaluated in parentheses. The Mann–Whitney test was used to determine statistical differences between DP and DN_total_; *p* values considered statistically significant (*p* < 0.05) are shown in bold. DP, dual-positive; DN_total_, total double-negative; NA, not analyzed.

Comparative analyses between the DP (*n* = 15) and total double-negative (DN_total_) (*n* = 257) groups indicated that individuals with antibodies against both SLA and HSP70 *L. infantum* were older (*p* = 0.0430), presented lower baseline CD4^+^ T cell counts (*p* = 0.0208) and lower CD4^+^/CD8^+^ T cell ratios (*p* = 0.0282), as well as a tendency towards higher HIV-1 viral load levels (*p* = 0.1268) suggestive of an advanced stage of HIV-1 infection than individuals without *L. infantum* antibodies (Table 1). Indeed, a large proportion of DP individuals (10/15, 66.67%) had <200 CD4^+^ T cells/μL at the time of HIV-1 diagnosis, which is considered AIDS-defining.

### 3.3. Subpopulation analysis reveals virological and immunological markers associated with dual-positive *L. infantum* serology in PWH

To ensure specificity for *L. infantum* in the DP serological samples and to exclude the possibility of cross-reactivity due to polyclonal activation and hypergammaglobulinemia, anti-*Trypanosoma cruzi* IgG serology was performed. Samples demonstrating positivity for anti-*T. cruzi* IgG (*n* = 2) were excluded from subsequent analyses (Supplementary Figure S2). Next, we performed a subpopulation analysis by matching DN individuals to the DP group by gender, age, viral load and CD4^+^ T cell count. This strategy resulted in the establishment of a third group (DN_paired_) (Figure 1), thereby mitigating the possibility of bias arising from polyclonal activation, hypergammaglobulinemia, high viral load or low CD4^+^ T cell levels. Controlling for these parameters limited the potential for variations in the HIV-1 transcriptomic profile and the state of immune activation. No statistical differences were observed between the DP and DN_paired_ groups regarding sociodemographic and clinical parameters, which underscores the pairing strategy’s equity (Table 2).

**Table 2:** Epidemiological, virological, and immunological characteristics of PWH in Bahia (Brazil) between 2014 and 2,105, stratified according to DP and DN_paired_.

	DP (*n* = 14)	DN_paired_ (*n* = 26)	*p* value
Male, *n*	78.57% (11)	88.46% (23)	NA
Age, years	42 (29–51)	33 (26–43)	0.2224
HIV-1 viral load, copies/ml	147,580 (2,284-700,074)	76,298 (15,757-141,159)	0.4572
CD4+ T cell count, cell/μl	166 (91–450)	300 (128–472)	0.4113
CD8+ T cell count, cell/μl	1,008 (744–1,607)	995 (852–1,489)	0.7474
CD4+/CD8+ T cell ratio	0.21 (0.058–0.39)	0.29 (0.14–0.49)	0.3954
CD45+ T cell count, cell/μl	1,788 (1,262-3,054)	1,958 (1,628-2,585)	0.6641

Data are expressed as median values, interquartile ranges (IIQ) and numbers of individuals evaluated (N), or as proportions (%) with the number of individuals evaluated in parentheses. The Mann–Whitney test was used to determine statistical differences between DP and DN_paired_; *p* values considered statistically significant (*p* < 0.05) are shown in bold. DP, dual-positive; DN_paired_, total double-negative; NA, not analyzed.

### 3.4. Subpopulation analysis reveals virological and immunological markers associated with dual-positive *L. infantum* serology in PWH

To investigate possible associations between transmission clusters of a more-aggressive HIV subtype and worsen HIV clinical status, HIV-1 subtyping was performed in the DP and DN_paired_ subset to infer. After excluding samples with a viral load <1,000 copies/mL, both groups were pooled together (*n* = 19) and submitted to phylogenetic analysis using the jpHMM-HIV model (Supplementary Figure S3). Concerning subtype distribution, subtype B (63.16%, 12/19) was the most prevalent, followed by subtypes C (15.79%, 3/19), recombinant BF (15.79%, 3/19), and D (5.26%, 1/19). No statistical differences in subtype were observed between the DP and DN_paired_ groups (*p* = 0.67) (Supplementary Table S2). No transmission cluster was identified. HIV-1 subtyping showed no impact on *L. infantum* serology titers, viral load, T cell count, or any other host immunological marker evaluated, indicating that HIV-1 subtypes B, C, D, F1, and BF exhibit similar virological and immunological outcomes in HIV/*L. infantum* co-infection.

**Figure 4:**
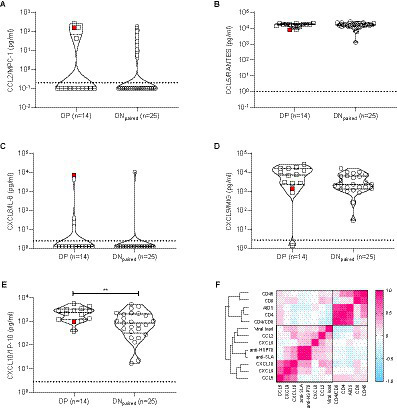
Chemokine profiles obtained from plasma/serum samples of DP and DN_paired_ groups. **(A)–(E)** Violin plots with dot-plot overlays illustrating chemokine plasma levels (pg/mL). Statistical comparisons made by the Mann–Whitney test in plasma levels (pg/mL) of CCL2/MPC-1 (*p* = 0.43), CCL5/RANTES (*p* = 0.94), CXCL8/IL-8 (*p* = 0.54), CXCL9/MIG (*p* = 0.061), and CXCL10/IP-10 (*p* = 0.0076) chemokines. Open squares (□): subgroup of double-positive PWH (positive for both anti-SLA and anti-HSP70 *L. infantum* antigens); open circle (○): subgroup of paired double-negative PWH (serologically negative for anti-SLA and anti-HSP70 *L. infantum* antigens). The red symbol represents the DP individual diagnosed with visceral leishmaniasis >20  years ago. **(F)** Heat map shows the correlation (pink: positive correlation; blue: negative correlation; white: no correlation) of epidemiological, virological and immunological characteristics of PWH.

Next, we quantified circulating cytokines and chemokines that might reflect an increase in systemic immune activation and have been previously associated with infection to *L. infantum* (Caldas et al., 2005; Peruhype-Magalhães et al., 2006; Frade et al., 2011; Carneiro et al., 2016). The systemic levels of cytokines tested (IL-1β, IL-6, IL-10, IL-12p70, and TNF) were below the detection limits in all samples (DP and DN_paired_ groups), concordant with our previous findings in a large Cuban cohort of PWH (Kouri et al., 2015). Conversely, most chemokines (except IL-8) were readily detected in most PWH, demonstrating a significant increase in CXCL10/IP-10 (*p* = 0.0076) and a tendency towards higher CXCL9/MIG levels (*p* = 0.061) (Figure 4) in the DP group compared to DN_paired_, suggesting increased immune activation in the group with DP serology for *L. infantum*.

**Figure 5:**
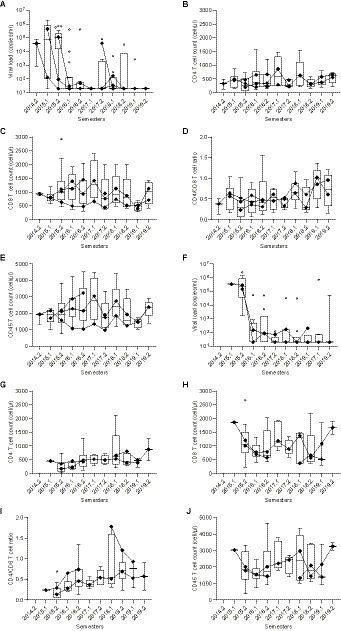
Long-term follow-up of CD4^+^, CD8^+^, CD4/CD8 ratio, CD45^+^, and viral load levels after therapy introduction in DP and DN_paired_ groups. **(A)–(J)** Box plots with line plot overlays connecting medians of CD4^+^, CD8^+^, CD4^+^/CD8^+^ ratio, CD45^+^ and viral load levels during follow-up. **(A)** and **(F)** Follow-up of viral load (copies/mL) in DN_paired_ **(A)** and DP **(F)** groups. **(B)** and **(G)** Follow-up of CD4^+^ T cell count (cells/μL) in DN_paired_ **(B)** and DP **(G)** groups. **(C)** and **(H)** Follow-up of CD8+ T cell count (cells/μL) in DN_paired_ **(C)** and DP **(H)** groups. **(D)** and **(I)** Follow-up of CD4^+^/CD8^+^ (ratio) in DN_paired_ **(D)** and DP **(I)** groups. **(E)** and **(J)** Follow-up of CD45^+^ T cell count (cells/μL) in DN_paired_ **(E)** and DP **(J)** groups.

This finding, together with the decreased CD4^+^ T cell counts found in our previous analysis (Table 2), corroborates the hypothesis that therapy-*naïve* HIV-1-infected subjects previously or currently infected with *L. infantum* exhibit a more intensified state of immune activation than HIV-1 positive, *L. infantum* seronegative subjects. During clinical follow-up, the introduction of antiretroviral therapy restored CD4^+^ T cell counts (median values of CD4^+^ T cell counts after 4–5 years of follow-up: DP = 620 cells/μL and DN_paired_ = 870 cells/μL) and reduced viral load levels to undetectable limits similarly in each group (Figure 5), with no statistically significant differences between groups (AUC comparison, Mann–Whitney test: VL: *p* = 0.6773; CD4^+^: *p* = 0.5239; CD8^+^: *p* = 0.6986; Ratio CD4^+^/CD8^+^: *p* = 0.9044; CD45^+^: *p* = 0.7540).

### 3.5. CXCL10 is shared between the HIV and visceral leishmaniasis gene signatures, while CXCL10, CXCL9, and CCL2 positively correlate with HIV viral load in untreated PWH

**Figure 6:**
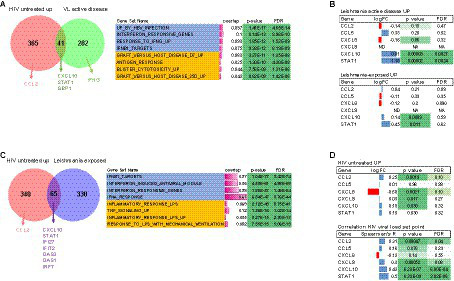
CXCL10 is shared between the HIV and visceral leishmaniasis disease signatures while CXCL10, CXCL9, and CCL2 transcripts positively correlate with HIV viral load in untreated PWH. Venn diagrams show overlap between **(A)** active disease gene signatures, i.e., genes upregulated in untreated PWH (Swiss cohorts) and visceral leishmaniasis patients (as compared to post-treatment) and **(C)** genes upregulated in untreated PWH and asymptomatic *Leishmania*-infected individuals (who did not develop disease). Systems biology analysis shows a significant enrichment of gene sets comprising immune activation and type I IFN signaling (right-hand panels). **(B)** Selective up-regulation of CXCL10 and STAT1 (as a positive control for IFN signaling) transcripts in both untreated visceral leishmaniasis patients (upper panel) and *L. infantum*-exposed individuals and **(D)** Selective up-regulation (upper panel) and positive correlation to viral load (lower panel) of CXCL10, CXCL9, and CCL2 and STAT1 (as a positive control for IFN signaling) transcripts in untreated PWH (Swiss cohort, *n* = 137).

Given the small number of immune activation markers and modest cohort size for the paired DP and DN groups, we aimed to validate and extend our findings by cross-examining independent cohorts of HIV-1 (Swiss HIV Cohort, *n* = 137) or Leishmania-infected individuals with and without active disease (Piaui Northeast Brazil, *n* = 30) with publicly available transcriptome-wide data (Rotger et al., 2010; Gardinassi et al., 2016). As shown in Figures 6A,B, a significant overlap (41 genes, enrichment *p* < 0.0001) was found between the HIV and visceral leishmaniasis gene signatures when comparing the top 500 transcripts significantly upregulated during active disease in both untreated HIV patients and untreated visceral leishmaniasis patients, as compared to their paired post-treatment groups. A systems biology analysis of the 41 genes shared by HIV and leishmaniasis active disease signatures revealed several significantly enriched biological processes, encompassing immune activation broadly and, more specifically, type I IFN signaling and antiviral response. The overlapping genes included CXCL10, which we found to be overexpressed in the DP group of our prospective cohort, as well as STAT1, the major transcription factor activated by IFN signaling. As detailed in Figure 6A and Supplementary Tables S3–S5, enriched gene sets from the Molecular Signatures Database (MSigDb) representing ‘immune activation’ included graft-versus-host disease, antigen recognition and blister cytotoxicity, while enriched gene sets representing ‘type I IFN/antiviral response’ comprised those upregulated in the liver by HBV infection, by IFN-alpha treatment of fibroblasts *in vitro* and by IFN-beta treatment of multiple sclerosis patients *in vivo*. Of interest, IFNG transcripts significantly decrease in monoinfected visceral leishmaniasis individuals, confirming our previous results obtained at the protein level for systemic IFN-γ in two independent VL cohorts (Caldas et al., 2005; Peruhype-Magalhães et al., 2006).

To define a specific ‘*Leishmania* infection’ gene signature, we used the top 500 significantly upregulated transcripts from *Leishmania*-infected individuals who did not develop disease, for which Leishmania infection was documented by a positive skin DTH reaction, as compared to matched DTH-negative individuals from the same endemic area (Gardinassi et al., 2016). Surprisingly, the overlap between the ‘untreated HIV’ and ‘*Leishmania*-seropositive (DP)’ gene signatures was even more pronounced than the overlap between the ‘untreated HIV’ and ‘untreated visceral leishmaniasis’ gene signatures, both by quantitative (65 shared genes, Figure 6B) and qualitative measures (median gene set overlap 9.4% vs. 3.2%, Mann–Whitney *p* < 0.0001, Supplementary Table S3), indicating that even asymptomatic *Leishmania* infection has a profound effect on the systemic immune response. Again, CXCL10 and STAT1 were shared among the two disease signatures (Figure 6B), and enriched gene sets included type I IFN signaling (genes upregulated by IFN-beta treatment of multiple sclerosis patients *in vivo* and by IFN-alpha treatment of fibroblasts *in vitro*) and immune activation (inflammatory response to LPS, TNF signaling).

Validation of our DP cohort results revealed that CXCL10, but not CCL2, CCL5 or CXCL8 transcripts, were significantly upregulated by *Leishmania infantum* infection with or without clinically definite visceral leishmaniasis (Figure 6C upper and lower panel). In the Swiss HIV Cohort, we found that CXCL10, CCL2 and, to a lesser extent, CXCL9 transcripts were significantly upregulated during untreated HIV-1 infection. In contrast, no significant effect was observed for CXCL8 in either asymptomatic or symptomatic *Leishmania infantum* infection, supporting our negative findings for CXCL8 genetic variation in visceral leishmaniasis patients from the same endemic region (Frade et al., 2011). However, CXCL8 transcripts were significantly downregulated during untreated HIV-1 infection (Figure 6D upper panel). Finally, as shown in Figure 6D (lower panel), CCL2, CXCL9, and CXCL10 transcripts were significantly correlated (even after stringent correction for genome-wide testing) with set point viral load in untreated PWH from the Swiss HIV Cohort (*n* = 137), confirming the results from our prospective cross-sectional cohort (Figure 4F).

## 4. Discussion

The geographic overlap of HIV-1 and *L. infantum* infections raises the possibility of co-infection, which several groups have shown to increase morbidity and mortality (Coura-Vital et al., 2014; Lima et al., 2017, 2018; Viana et al., 2017; Diniz et al., 2018; Fontoura et al., 2018; Luz et al., 2018; Rezaei et al., 2018; Sousa et al., 2018; Botana et al., 2019; Diro et al., 2019; Horrillo et al., 2019; Maia-Elkhoury et al., 2019; Prestes-Carneiro et al., 2019; Santos et al., 2019; Silva de Lima et al., 2019; van Griensven et al., 2019). Several groups have studied the extent of this overlap in several Brazilian regions. However, no studies have attempted to determine the seroprevalence of *L. infantum* among PWH in the state of Bahia, despite the significant prevalence of both infections (Secretaria de Saúde do Estado da Bahia, 2022a, 2022b). We used a two-stage cross-sectional cohort approach (CSC-I, *n* = 5,346 and CSC-II, *n* = 317) and identified positive serology for *L. infantum* ranging from 4.73% (DP for anti-SLA and anti-HSP70) to 18.93% in the CSC-II (union of all positive samples for anti-SLA and anti-HSP70), with an intermediate seroprevalence for only anti-SLA of 16.27% in the CSC-I and 15.46% in the CSC-II.

The observed frequencies of *Leishmania* seroprevalence in PWH using any of the serology strategies in both the cross-sectional cohorts support previous findings falling within the range of what has already been described in other Brazilian regions – Ceará: 5.4–6.6% (Távora et al., 2015); Distrito Federal: 16.0% (Carranza-Tamayo et al., 2009); Maranhão: 4.2% (Carvalho et al., 2013); Mato Grosso: 9.9% (Luz et al., 2018); Mato Grosso do Sul: 5% (Botelho and Natal, 2009); Pernambuco: 5.6% (Sousa et al., 2018); Piauí: 8.2% (Soares et al., 2008); Rio Grande do Norte: 4.9% (Lima et al., 2017); Sergipe: 4.5% (Santos et al., 2019); and Tocantins: 2.1% (Albuquerque et al., 2014). Moreover, demographic data indicated that all DP patients were born in the macroregions surrounding the cities of Salvador and Feira de Santana, which are responsible for 33.8% (1,074/3,172) of notified VL cases in Bahia between 2007 and 2015 (Secretaria de Saúde do Estado da Bahia, 2018).

The observed discrepancy between the results obtained with the two different immunoassays might be explained by the rigor employed to determine cutoffs for each serological test and the imbalance in protein fractions between these two antigen-based diagnostic methods. SLA is a heterogeneous mix of antigens and has a lower concentration of HSP70 than parasite structural proteins (actin, tubulin, etc.). Even though it will pick up a broader set of samples, some samples with low antibody levels will fail. On the other hand, the recombinant HSP70 antigen is homogenous and highly concentrated and has a better detection limit with better sensitivity than other recombinant proteins (rH2A, rH2B, rH3, rH4, and rKMP11) and high specificity when evaluated against sera from patients with Chagas’ disease, Tuberculosis, Leprosy or Systemic Lupus Erythematosus (Souza et al., 2013). We considered only those samples demonstrating dual positivity to increase confidence regarding past or present *L. infantum* infection. This strategy was underscored by the identification of one individual with a previous history of VL in the DP group and no cases with an earlier history of VL in the DN_paired_ group. Furthermore, we confirmed that no statistical differences regarding epidemiological, virological, and immunological characteristics were observed comparing DP, SLA, HSP70 and HSP70^+^SLA groups.

Unfortunately, only serum or plasma samples were available for HIV-1 RNA extraction, thus precluding DNA analysis to detect *L. infantum* by qPCR, which we acknowledge as one of the limitations of our study. However, Fukutani et al. (2014) demonstrated a concordance of 68% between anti-SLA serology and PCR, and found a 5.40% rate of ongoing asymptomatic *L. infantum* infection among blood donors in Salvador, Bahia. Of note, this concordance between PCR and serology could be compromised by the reduced serology sensitivity (66%) to identify true positive ongoing *Leishmania*-infected cases among PWH. In addition, we also highlight as a limitation the absence of Leishmanin skin test in our study (DTH) to access cellular immune response present during *Leishmania* infection, which is more sensitive and specific to determine ongoing asymptomatic infection than serological assays (Botana et al., 2019). Nevertheless, positive IgG serology for *L. infantum* indicates immunological memory of at least a previous *Leishmania* infection before HIV diagnosis. Strikingly, we identified a DP individual with a pronounced humoral response two decades after a VL clinical diagnosis. This ongoing humoral response most likely reflects chronic antigen exposure, emphasizing the concept that *Leishmania* infection is lifelong in most patients, as well as murine models of disease (Bogdan et al., 2000; Seyed et al., 2018; Paduch et al., 2019). A wealth of studies worldwide (Oryan and Akbari, 2016; Grande et al., 2017; Zacarias et al., 2017; Bruhn et al., 2018; Guedes et al., 2018; Luz et al., 2018) and a recent systematic review (Fontoura et al., 2018) have demonstrated a high frequency of relapse of VL in PWH, as well as increased mortality. Relapse of VL has been reported after anti-leishmaniasis treatment, even years after the patient was considered clinically cured, especially in PWH and/or immunosuppressive conditions (Horrillo et al., 2019; Silva de Lima et al., 2019).

Prior large studies, including from our group, have found striking differences in the prevalence of VL within different regions in Brazil, as well as Southern Europe. The highest seroprevalence was observed in Mato Grosso, 41.40% of blood donors, in contrast to other regions in Brazil [Salvador: 5.40% of 700 (Fukutani et al., 2014); Paraná: 11.40% of 176 (Braga et al., 2015); Fortaleza: 17.10% of 431 (Monteiro et al., 2016)], and in Southern Europe [France: 13.40% of 565 (le Fichoux et al., 1999); Spain: 3.10% of 1,437 (França et al., 2018)].

Studies conducted in populations of newly diagnosed HIV-1-infected individuals from regions endemic for leishmaniasis in Brazil reported a prevalence of HIV-1/*L. infantum* co-infection ranging from 4.20 to 20.20%. In these studies, *L. infantum* infection was determined by parasitological, serological and/or molecular testing (Soares et al., 2008; Carranza-Tamayo et al., 2009; Orsini et al., 2012; Carvalho et al., 2013). However, in co-infection studies focused on VL patients, which were diagnosed by parasitological, molecular or immunofluorescence methods, the prevalence of HIV-1/*L. infantum* coinfection ranged from 36.60 to 55.60% (Souza et al., 2012; Cota et al., 2014; Druzian et al., 2015).

The present study also investigated possible differences in the prevalence of HIV-1 variants associated with *L. infantum* infection in PWH. With these subtyping results, we would like to investigate if a possible worsen disease progression in PWH with positive serology for *L. infantum* cases was related to cluster of transmission of a more-aggressive HIV subtype (e. g., HIV Subtype D) (Khouri and Vandamme, 2015). However, no differences were found concerning HIV-1 isolates between matched samples of patients infected (DP) or not (DN_paired_) to *L. infantum*. Within these isolates, subtype B (63.16%) was found to be the most prevalent, followed by C (15.79%), recombinant BF (15.79%) and D (5.26%). Recent studies in Bahia that evaluated HIV-1 variant distribution have described the wide distribution of subtype B, followed by a smaller proportion of recombinant BF units (Araujo et al., 2010; Monteiro-Cunha et al., 2011; Santos et al., 2011). Of note, the higher prevalence of subtype C (15.79%) found herein stands in contrast to other studies also conducted in this region, which reported a very low prevalence ranging from 1.70 to 2.50% (Chavez et al., 2007; Santos et al., 2009; Monteiro-Cunha et al., 2011).

Moreover, our confirmation of the circulation of subtype D in the state is following work by Monteiro et al. (2009), who previously reported the presence of subtype D in individuals from Feira de Santana (0.60% of _pol_F/_env_D subtype), a city located ~100 km from Salvador. It is worth noting that the subtyping analysis performed herein involved three different methodologies (REGA HIV-1 Subtyping Tool, phylogeny and jpHMM-HIV probabilistic modeling), which lends support to the presently reported distribution. Furthermore, our subtype identification was performed using HIV-1 pol sequences, inferring subtype frequencies can vary when complete genome sequencing is performed.

Prior research into host immune responses has demonstrated that *L. infantum* infection induces a higher level of immune activation in co-infected PWH, which could lead to a more rapid progression to AIDS (Ipp et al., 2014; Claiborne et al., 2015; Hoffmann et al., 2016; Utay and Hunt, 2016). Our study found a roughly five-fold increase in circulating CXCL10/IP-10 and CXCL9/MIG chemokines in PWH exposed to *L. infantum*, supporting a link between positive serology for *L. infantum* and immune activation, which we validated at the transcript level for CXCL10 in independent HIV-1 and Leishmania-infected cohorts. These chemokines are strongly induced by both type I (IFN-α/β) and type II IFN (IFN-γ) (Kedzierska and Crowe, 2001; Mehla, 2013; Kenway-Lynch et al., 2014; Utay and Douek, 2016). It has been previously reported that Th1 subpopulations of CD4^+^ CD95^+^ T cells in HIV-1 infected individuals are highly susceptible to HIV-1 envelope gp120-induced apoptosis (Accornero et al., 1997; Cummins and Badley, 2010), which may corroborate the deleterious role of intensified immune activation (Costa et al., 2012; Sun et al., 2015; Rodrigues et al., 2016). In a submitted manuscript (Khouri et al.), we demonstrated an increase in both systemic soluble Fas as well as membrane-bound Fas/CD95^+^ expression in CD8^+^ cells in HIV/VL co-infection, as compared to HIV-1 or VL mono-infected groups, in a large cohort of individuals recruited during an outbreak of VL in Piaui, another state in the same northeast region of Brazil. Since we previously demonstrated STAT1-mediated IFN signaling as a preferential inducer of Fas/CD95^+^ at both the protein and transcript level (Farre et al., 2008; Khouri et al., 2018), this study as well as Khouri et al. (submitted) underscore the pivotal role of IFN-driven immune activation in HIV-1 and *Leishmania* infantum co-infection and its deleterious effect on both AIDS progression and visceral leishmaniasis.

The present study aimed to describe the prevalence and consequences of *L. infantum* infection in a population of antiretroviral treatment-*naïve* PWH at diagnosis. We found a high seroprevalence of *L. infantum* in treatment-*naïve* PWH in Bahia (Brazil) associated with a more intensified state of immune activation as compared to HIV-1 mono-infected subjects, possibly exacerbating progression to AIDS, which was observed in 66.67% in co-infected individuals at diagnosis. This immune activation and disease progression in PWH is reversed by immediate ART implementation (Buzón et al., 2010). Brazil adopted therapy as prevention (TasP) for HIV in 2013, which through our National Health System (SUS) offers and treats every HIV diagnosed patient in the country, independent of the CD4^+^ T cell level. However, in Brazil, Visceral Leishmaniasis cases are investigated only when severe VL-like symptoms are already present, precluding ongoing asymptomatic or mild visceral leishmaniasis diagnosis. Thus, Leishmania-infected and ART-*naïve* PWH with low CD4^+^ T cells (<200 cell/μL) and high viral load are at high risk of developing severe visceral leishmaniasis (Viana et al., 2017). These findings underscore the urgent need to increase awareness and define public health strategies for the management and prevention of HIV-1 and *L. infantum* co-infection.

## Data availability statement

The data presented in this study have been deposited in the NCBI GenBank under accession numbers MW596907, MW596966, MW597006, MW597017, MW597019, MW597022, MW597023, MW597030, MW597036, MW597044, MW597056, MW597059–MW597061, MW597063, MW597068, MW597073–MW597075.

## Ethics statement

The studies involving human participants were reviewed and approved by Institutional Review Board of the Gonçalo Moniz Institute (IGM-FIOCRUZ) (protocol number 1.764.505). The patients/participants provided their written informed consent to participate in this study.

## Author contributions

RK and JW contributed to conception, design of the study and funding acquisition. LM, MP, FT, MBS, AT, and TV contributed to data curation. LM, LS, LA, CO, and LR performed formal analysis. MOS, AR, IS, JS, DC, CC, and MG were responsible for the investigation. RK and JW developed the methodology. AB, A-MV, JV, MS, MOS, AR, DC, and CC provided resources. JW, RK, and LM contributed to visualization. LM and RK wrote the first draft of the manuscript. RK, A-MV, JV, MB-N, AB, LS, LA, VB, MS, BA, KA, and CO wrote sections of the manuscript. All authors contributed to manuscript revision, read, and approved the submitted version.

## Funding

This study was financed in part by the Coordenação de Aperfeiçoamento de Pessoal de Nível Superior – Brasil (CAPES) – Finance Code 001, RK; Fundação de Amparo à Pesquisa do Estado da Bahia (FAPESB, grant APP0032/2016, RK); Brazilian National Council for Scientific and Technological Development (CNPq, grant 65083/2015-8, LS); FAPESB/CNPq (008/2014 PRONEX grant 8111/2014, AB); Fonds voor Wetenschappelijk Onderzoek Vlaanderen (grant G0D6817N, A-MV and JW). None of the funding organizations had any role in the study design, data collection, data interpretation or writing of this report.

## Conflict of interest

The authors declare that the research was conducted in the absence of any commercial or financial relationships that could be construed as a potential conflict of interest.

## Publisher’s note

All claims expressed in this article are solely those of the authors and do not necessarily represent those of their affiliated organizations, or those of the publisher, the editors and the reviewers. Any product that may be evaluated in this article, or claim that may be made by its manufacturer, is not guaranteed or endorsed by the publisher.

## Footnotes

^1^ https://www.genomedetective.com/app/typingtool/hiv

^2^ https://www.hiv.lanl.gov/content/sequence/NEWALIGN/align.html
