# Supplementary material for: High seroprevalence of Leishmania infantum is linked to immune activation in people with HIV: a two-stage cross-sectional study in Bahia, Brazil
Source: Front Microbiol. 2023 Jul 19;14:1221682. doi: 10.3389/fmicb.2023.1221682 (PMC10436095; doi:10.3389/fmicb.2023.1221682)

Supplementary Material

High seroprevalence of Leishmania infantum is linked to immune activation in people with HIV: a two-stage cross-sectional study in Bahia, Brazil

Laise de Moraes^1,2^, Luciane Amorim Santos^1,2,3^, Liã Bárbara Arruda^4^, Maria da Purificação Pereira da Silva^5^, Márcio de Oliveira Silva^5^, José Adriano Góes Silva^1,2,5^, André Ramos^5^, Marcos Bastos dos Santos^2^, Felipe Guimarães Torres^2^, Cibele Orge^2^, Antonio Marcos dos Santos Teixeira^2^, Thiago Santos Vieira^2^, Laura Ramírez^6^, Manuel Soto^6^, Maria Fernanda Rios Grassi^2,3^, Isadora Cristina de Siqueira^2^, Dorcas Lamounier Costa^7^, Carlos Henrique Nery Costa^7^, Bruno de Bezerril Andrade^2,3^, Kevan Akrami^1,2^, Camila Indiani de Oliveira^1,2,3^, Viviane Sampaio Boaventura^1,2,8^, Manoel Barral-Netto^1,2^, Aldina Barral^1,2*^, Anne-Mieke Vandamme^9,10*^, Johan Van Weyenbergh^9*^, Ricardo Khouri^1,2,9^

*** Correspondence:** Corresponding Author: [ricardo.khouri@fiocruz.br](mailto:ricardo.khouri@fiocruz.br)

**Supplementary Figure 1.** Sensitivity and specificity of anti-*L. infantum* IgG assays


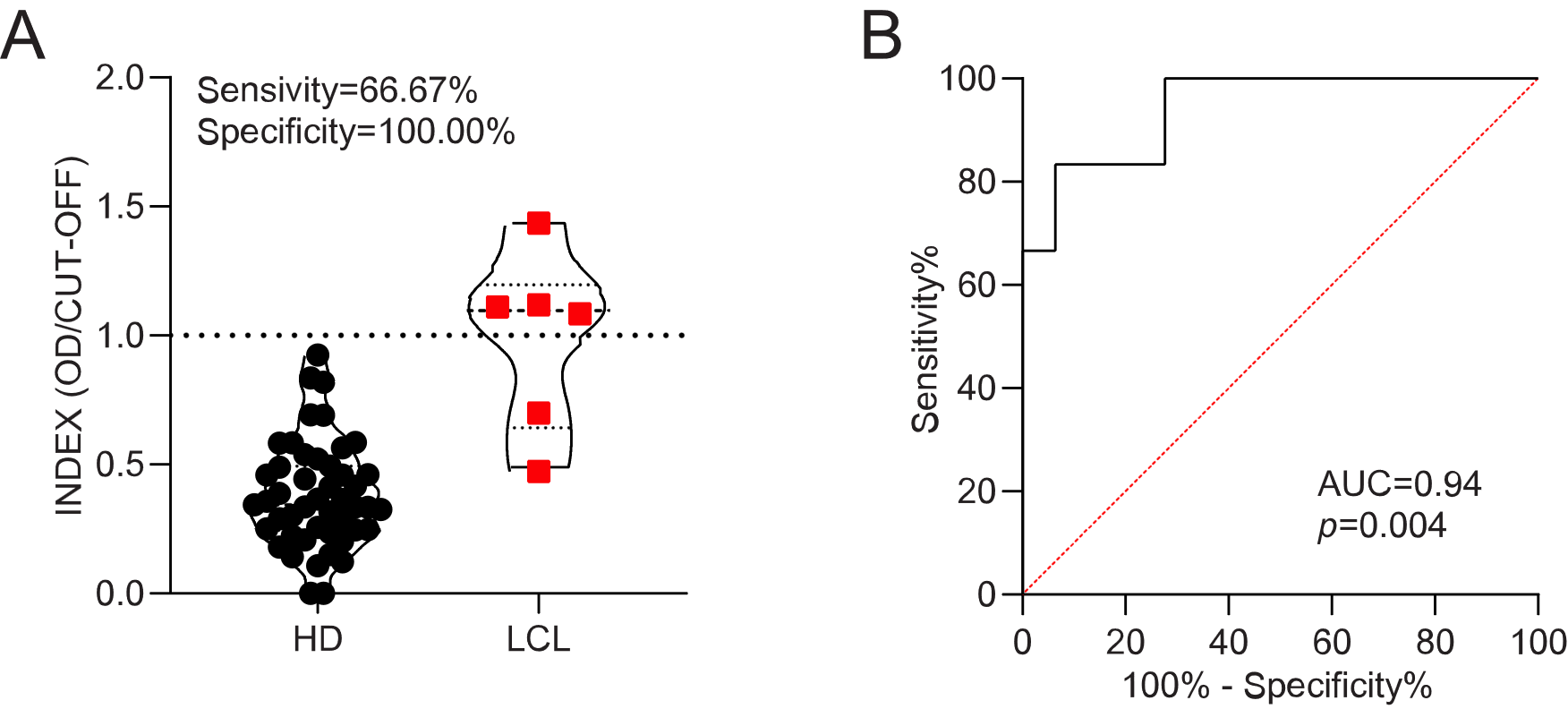


**Supplementary Figure 2.** Detection of anti-*Trypanosoma cruzi* IgG antibodies in plasma/serum samples of DP and DN_paired_ groups


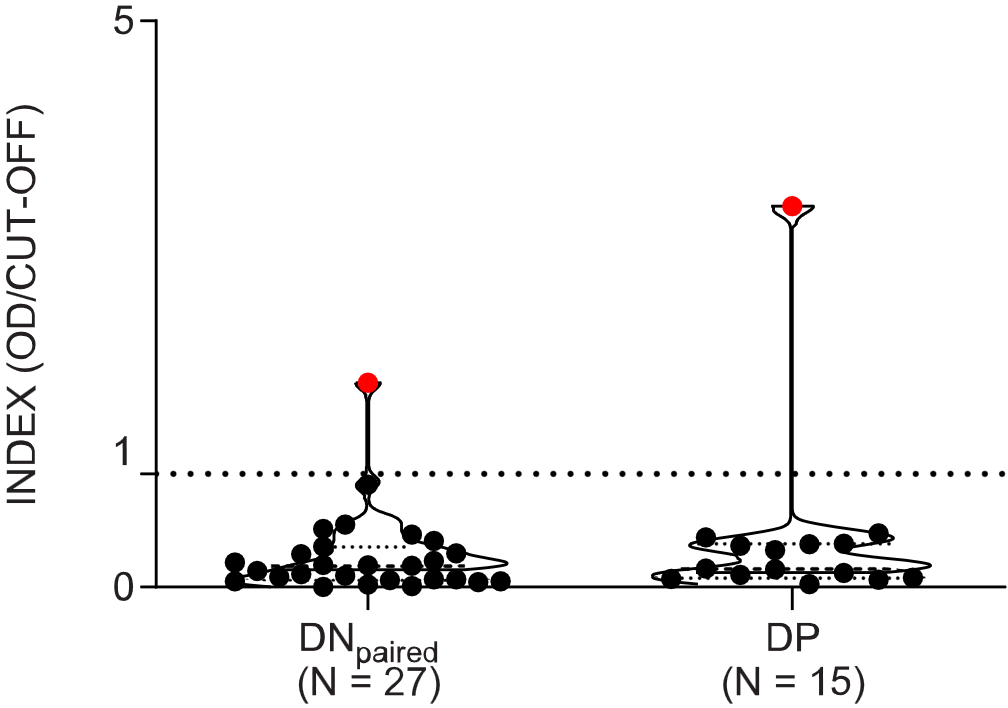


**Supplementary Figure 3.** Phylogenetic analysis of all *pol* HIV-1 pure subtypes


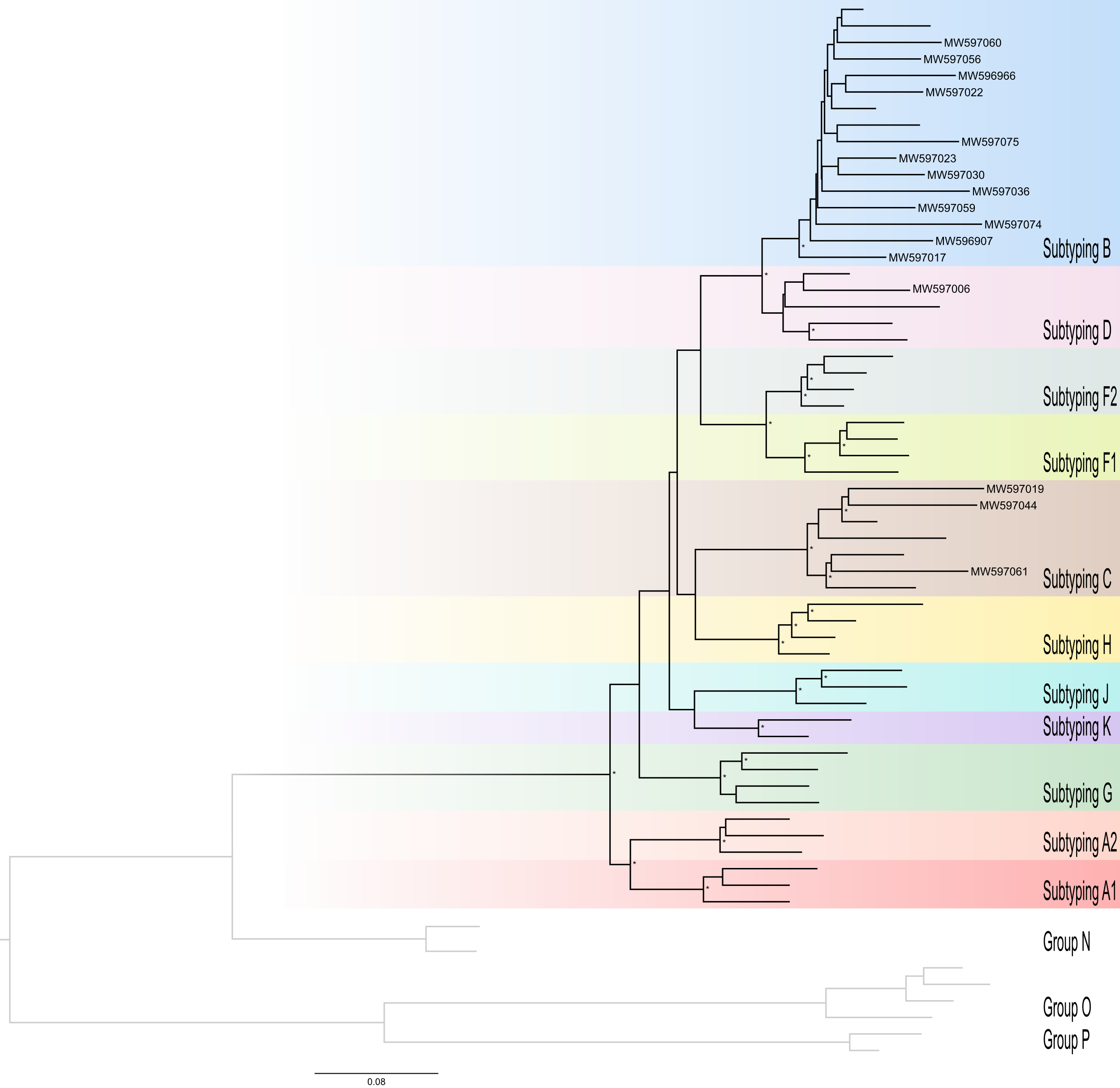

Supplement: Supplementary file 1 [file Table_1.DOCX]
